# The feasibility of pragmatic influenza vaccine randomized controlled real-world trials in Denmark and England

**DOI:** 10.1038/s41541-022-00444-6

**Published:** 2022-02-23

**Authors:** Joshua Nealon, Daniel Modin, Rebecca E. Ghosh, Deborah Rudin, Gunnar Gislason, Helen P. Booth, Jens Ulrik Stæhr Jensen, Rachael Williams, Hilary Shepherd, Eleanor Yelland, Helene Bricout, Sandra S. Chaves, Tor Biering-Sørensen

**Affiliations:** 1 Sanofi Pasteur Medical Evidence Generation, Lyon, France; 2 School of Public Health, Li Ka Shing Faculty of Medicine, The University of Hong Kong, Pokfulam, Hong Kong Special Administrative Region, Pokfulam, China; 3 Department of Cardiology, Copenhagen University Hospital—Herlev and Gentofte, Copenhagen, Denmark; 4 Clinical Practice Research Datalink, Medicines and Healthcare products Regulatory Agency, London, UK; 5 Sanofi Pasteur Global Medical Affairs, Swiftwater, PA USA; 6 Department of Pulmonology, Copenhagen University Hospital—Herlev and Gentofte & Department of Biomedical Sciences, Faculty of Health and Medical Sciences, University of Copenhagen, Copenhagen, Denmark; 7 Modelling, Epidemiology and Data Science, Sanofi Pasteur, Lyon, France; 8 Department of Biomedical Sciences, Faculty of Health and Medical Sciences, University of Copenhagen, Copenhagen, Denmark

**Keywords:** Randomized controlled trials, Inactivated vaccines, Influenza virus, Policy and public health in microbiology

## Abstract

We estimated the frequency of non-specific influenza-associated clinical endpoints to inform the feasibility of pragmatic randomized controlled trials (RCT) assessing relative vaccine effectiveness (rVE). Hospitalization rates of respiratory, cardiovascular and diabetic events were estimated from Denmark and England’s electronic databases and stratified by age, comorbidity and influenza vaccination status. We included a seasonal average of 4.5 million Danish and 7.2 million English individuals, 17 and 32% with comorbidities. Annually, approximately 1% of Danish and 0.5% of English individuals were hospitalized for selected events, ~50% of them respiratory. Hospitalization rates were 40–50-fold and 2–10-fold higher in those >50 years and with comorbidities, respectively. Our findings suggest that a pragmatic RCT using non-specific endpoints is feasible. However, for outcomes with rates <2.5%, it would require randomization of ~100,000 participants to have the power to detect a rVE difference of ~13%. Targeting selected groups (older adults, those with comorbidities) where frequency of events is high would improve trial efficiency.

## Introduction

The World Health Organization (WHO) recommends annual vaccination as the most effective method to prevent influenza^1^. Randomized control trials (RCTs) have, in recent years, relied on laboratory confirmed influenza as a study endpoint to demonstrate the efficacy of influenza vaccines^2^. However, there is a growing body of evidence that influenza is associated with a broader spectrum of non-respiratory events including cardiovascular, neurological and other complications^3–5^. If influenza virus infection precipitates these events, vaccination should be expected to prevent a proportion of them^6^. Limited evidence of the value of influenza vaccination at preventing non-respiratory outcomes exists based on observational studies, meta-analysis of RCT data and reanalysis of study safety data^7–9^.

RCTs are the most valid study designs to demonstrate causal relationships^10^; but including non-specific endpoints in a traditional RCT may require large sample sizes because only a proportion of captured non-respiratory events would be influenza-associated [and therefore vaccine-preventable]. Results would also be sensitive to unpredictable and poorly-understood, time-lagged relationships between influenza and related outcomes or complications which could ‘dilute’ vaccine efficacy/effectiveness (VE)^11^. Differentiated influenza vaccines have demonstrated improved immunogenicity and protection for older adults and some studies have included non-specific cardiovascular or other secondary events to illustrate the full public health value of these vaccines as compared with traditional influenza vaccines^12–15^. Studies comparing two vaccines measure *relative* VE (rVE) which, using an efficacious comparator, report smaller effect sizes and therefore require even larger sample sizes to demonstrate superior protection from influenza-associated cardiovascular and non-respiratory events.

Pragmatic trials, which typically measure outcomes using real world data from existing databases or public health registers, may be a feasible method of randomizing interventions in hundreds of thousands of study participants and therefore increase power to measure rVE against non-specific outcomes in a cost-effective manner^16,17^. Their sample sizes are dictated by the estimated rVE and incidence rate of the outcome under assessment. These studies would be feasible if it is logistically and financially possible to randomize and vaccinate the necessary sample size within a single or multiple healthcare systems from which outcomes could be reliably captured.

We conducted a retrospective study using electronic medical records from Denmark and England to estimate the incidence rate of cardiovascular, respiratory and exacerbation of diabetes events in adults ≥18 years, hospitalized during influenza season, to guide future pragmatic RCTs exploring broader, clinically-important, influenza-associated endpoints. We then estimated the sample size requirements to conduct a pragmatic RCT under different population inclusion and rVE scenarios and discussed the feasibility of conducting such studies.

## Results

### Demographics and influenza vaccination coverage rate

**Fig. 1:**
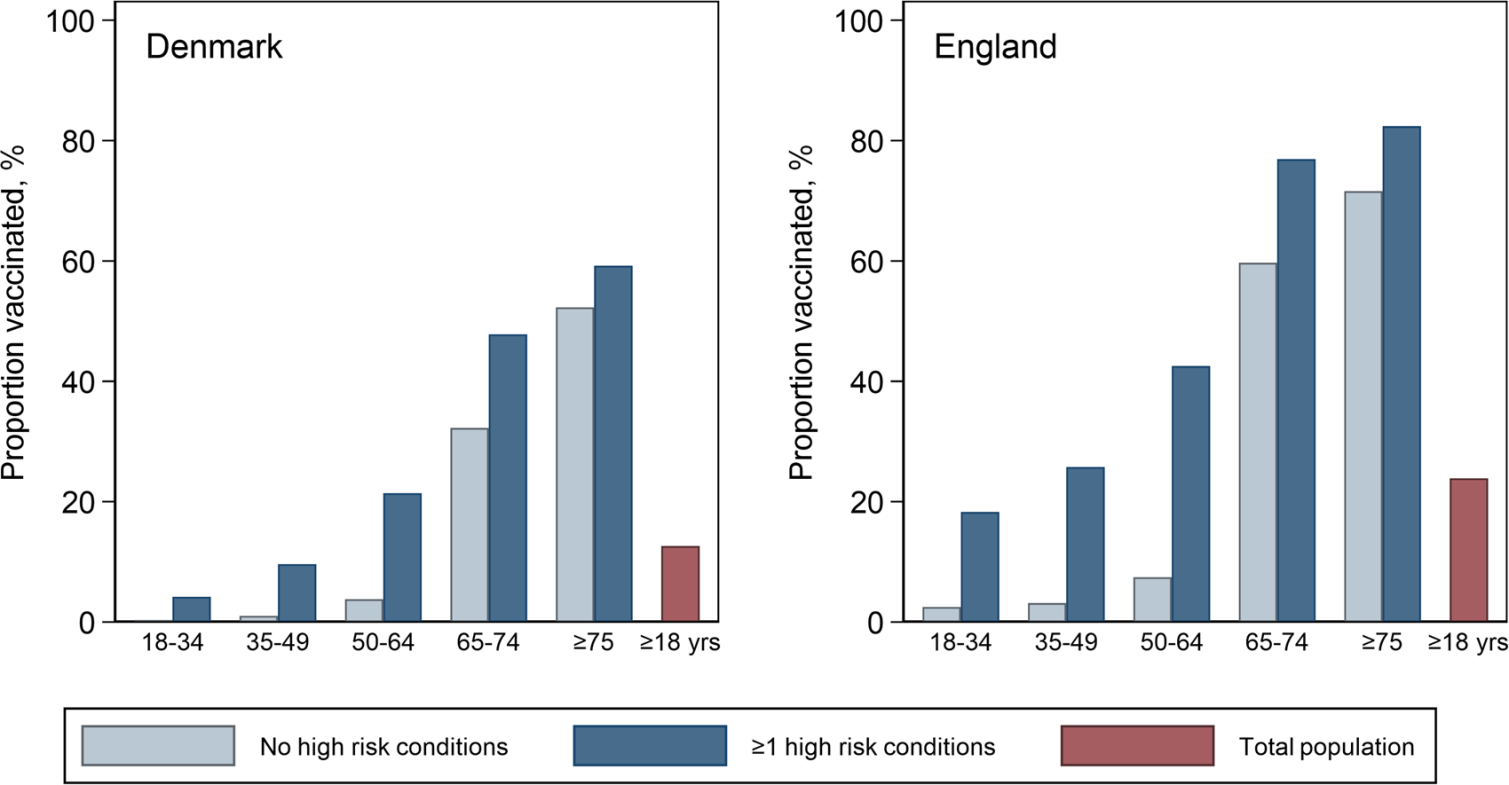
Annual average influenza vaccination coverage rates by age group in individuals with and without high risk conditions from Denmark and England. Individuals with record of influenza vaccination between August 1st and Jan 31st of each season from Denmark (2010/11–2017/18) and England (2010/11–2018/19).

From Denmark, the study cohort aged ≥18 included a seasonal average of 4,469,268 individuals, 50.7% female. Overall, 17% of the study population had ≥1 high risk condition, increasing from 5.7% in those 18–34 years to 43% in those ≥75 years (Table 1). From England, the cohort included a seasonal average of 7,212,471 people of whom 50.5% were female. In the overall population, 32% had ≥1 high risk condition, increasing from 16.7% in the 18–34 years to 63% in those ≥75 years. Cardiovascular, diabetes, immunocompromised, asthma and other respiratory conditions were the most common high-risk conditions in both countries. Influenza vaccination coverage rates (VCR) captured in these healthcare databases was 13% in Denmark and 24% in England, increasing in the populations aged ≥75 to 55 and 78%, respectively (Fig. 1; Supplementary Fig. 1). VCR was 6–10 fold higher in people with high-risk conditions vs those without in populations aged <65. This difference was much smaller in older adults: in Denmark 59% of adults aged ≥75 with high-risk conditions were vaccinated vs 52% of those without; in England the corresponding proportions were 82 and 72% (Fig. 1 and Supplementary Table 3).

**Table 1:** Seasonal average study population (#) and percentage (%) by age group and high-risk conditions, Denmark (2010/11 to 2017/18) and England (2010/11–2018/19).

		Denmark					England				
Age group:		18–34 yrs	35–49 yrs	50–64 yrs	65–74 yrs	>75 yrs	18–34 yrs	35–49 yrs	50–64 yrs	65–74 yrs	>75 yrs
Study population	#	1,184,338	1,162,715	1,091,518	607,953	422,744	2,124,434	1,932,551	1,641,127	810,820	703,539
No high-risk condition	#	1,116,222	1,048,161	888,877	418,772	240,462	1,770,594	1,451,475	1,031,906	395,988	259,218
	%	94.3	90.1	81.4	68.9	57.0	83.3	75.1	62.9	48.8	36.8
Any high-risk condition	#	68,116	114,554	202,642	189,182	182,282	353,840	481,076	609,221	414,832	444,320
	%	5.7	9.9	18.6	31.1	43.0	16.7	24.9	37.1	51.2	63.2
Asthma	#	9424	10,298	9990	5943	4181	149,687	152,166	137,866	73,471	61,578
	%	0.8	0.9	0.9	1.0	1.0	7.0	7.9	8.4	9.1	8.8
Respiratory disorders	#	1303	4329	15,975	20,205	24,617	1716	7642	37,639	48,471	52,624
	%	0.1	0.4	1.5	3.3	5.8	0.1	0.4	2.3	6.0	7.5
Cardiovascular	#	3959	17,050	58,372	72,192	95,037	4812	18,310	74,855	97,262	168,364
	%	0.3	1.5	5.3	11.9	22.4	0.2	0.9	4.6	12.0	23.9
Diabetes	#	10,027	26,678	69,683	67,618	51,795	19,534	56,832	137,204	116,557	121,541
	%	0.8	2.3	6.4	11.1	12.2	0.9	3.0	8.4	14.4	17.3
Endocrine disorders*	#	7455	14,029	16,489	10,800	10,628	18,516	38,451	52,165	33,277	35,860
	%	0.6	1.2	1.5	1.8	2.5	0.9	2.0	3.2	4.1	5.1
Blood disorders	#	476	488	716	953	1476	3584	2914	1591	696	787
	%	0.0	0.0	0.1	0.2	0.4	0.2	0.2	0.1	0.1	0.1
Immunocompromised	#	11,361	23,440	47,006	51,182	45,635	32,523	37,212	54,186	53,220	62,564
	%	1.0	2.0	4.3	8.4	10.7	1.5	1.9	3.3	6.6	8.9
Kidney disorders	#	840	1,918	4046	5792	9513	1735	6964	26,055	46,062	100,293
	%	0.1	0.2	0.4	0.9	2.2	0.1	0.4	1.6	5.7	14.2
Liver disorders	#	1849	4276	6517	3043	1177	3226	10,620	16,654	7704	3319
	%	0.2	0.4	0.6	0.5	0.3	0.2	0.6	1.0	0.9	0.5
Neurological disorders	#	1498	3730	5462	5748	13,106	1331	3924	7279	11,354	52,003
	%	0.1	0.3	0.5	0.9	3.1	0.1	0.2	0.4	1.4	7.4

*Excludes diabetes.

### Outcome rates in vaccinated and unvaccinated individuals

A total of 375,870 hospitalizations for any study outcome were captured over eight influenza seasons in Denmark and 325,799 hospitalizations over nine seasons in England (Table 2). Hospitalization rates varied by age group in Denmark from a low of 119 (95% CI: 117; 121) per 100,000 population in the 18–34 years age group, increasing ~17-fold to 2037 (2024; 2050) in the 65–74 year age group and ~40-fold to 4772 (4749; 4796) in the ≥75 years age group. In England rates in those aged 18–34 were 49 (48–50) per 100,000; 1013 (1006; 1021) (21-fold higher) in those 65–74 years old and 2,545 (2532; 2557) (~52x higher) in those aged ≥75 years.

**Table 2:** Seasonal average number (#) and incidence rates (IR) of selected hospitalized outcomes per 100,000 population by age group for the total study population, Denmark (from 2010/11–2017/18) and England (from 2010/11–2018/19).

Age group:		18–34 yrs	35–49 yrs	50–64 yrs	65–74 yrs	≥75 yrs	All ages
**Denmark**							
Any hospitalization*	#	1414	3350	9659	12385	20177	46984
	IR	119 (113; 125)	288 (282; 294)	885 (872; 898)	2037 (1995; 2080)	4773 (4630; 4919)	1051 (1021; 1081)
**Respiratory hospitalizations**	#	961	1741	4596	6432	11754	25484
	IR	81 (75; 87)	150 (143; 157)	421 (406; 437)	1058 (1002; 1117)	2780 (2603; 2970)	570 (535; 606)
Influenza + pneumonia	#	616	1137	2709	3824	8109	16395
	IR	52 (45; 60)	98 (91; 105)	248 (238; 258)	629 (586; 675)	1918 (1768; 2081)	367 (340; 395)
Influenza	#	97	118	164	165	260	804
	IR	8.2 (5.5; 12)	10 (7; 14)	15 (9; 24)	27 (14; 52)	62 (28; 136)	18 (10; 31)
**Cardiovascular hospitalizations**	#	203	1095	3410	3993	6320	15022
	IR	17 (16; 18)	94 (90; 98)	312 (307; 318)	657 (633; 681)	1495 (1417; 1577)	336 (329; 343)
**Diabetic hospitalizations**	#	154	126	155	154	200	789
	IR	13 (12; 14)	11 (10; 12)	14 (14; 15)	25 (24; 26)	47 (45; 50)	18 (17; 18)
**England**							
Any hospitalization*	#	1033	2401	6647	8215	36200	17904
	IR	49 (45; 52)	124 (118; 131)	405 (386; 425)	1013 (964; 1065)	2545 (2337; 2771)	502 (467; 539)
**Respiratory hospitalizations**	#	704	1282	3227	4404	19140	9523
	IR	33 (30; 37)	66 (61; 72)	197 (182; 213)	543 (495; 596)	1354 (1172; 1564)	265 (236; 298)
Influenza + pneumonia	#	348	702	1617	2356	11672	6649
	IR	16 (14; 20)	36 (32; 42)	99 (85; 114)	291 (246; 343)	945 (784; 1140)	162 (136; 192)
Influenza	#	66	89	151	139	688	241
	IR	3.1 (1.7; 5.9)	4.6 (2.5; 8.7)	9.2 (4.6; 18)	17 (8; 38)	34 (14; 85)	9.5 (4.6; 20)
**Cardiovascular hospitalizations**	#	202	1032	3367	3874	17173	8698
	IR	9.5 (8.8; 10)	53 (51; 56)	205 (199; 212)	478 (472; 484)	1236 (1202; 1272)	238 (231; 245)
**Diabetic hospitalizations**	#	131	111	149	125	804	288
	IR	6 (6; 7)	6 (5; 6)	9 (8; 10)	15 (14; 16)	41 (39; 43)	11 (11; 12)

95% confidence intervals (in brackets) from the Poisson distribution. *Any hospitalization for an outcome included in our analysis.

Influenza hospitalization rates varied between seasons in Denmark from 2 per 100,000 in the 2011/12 season to 52 per 100,000 in the 2017/18 season; and in England from 0.6 per 100,000 in 2011/12 to 33 per 100,000 in 2017/18. Inter-seasonal variation in cardiovascular or diabetic outcome groups was less pronounced, normally varying by <20%.

**Fig. 2:**
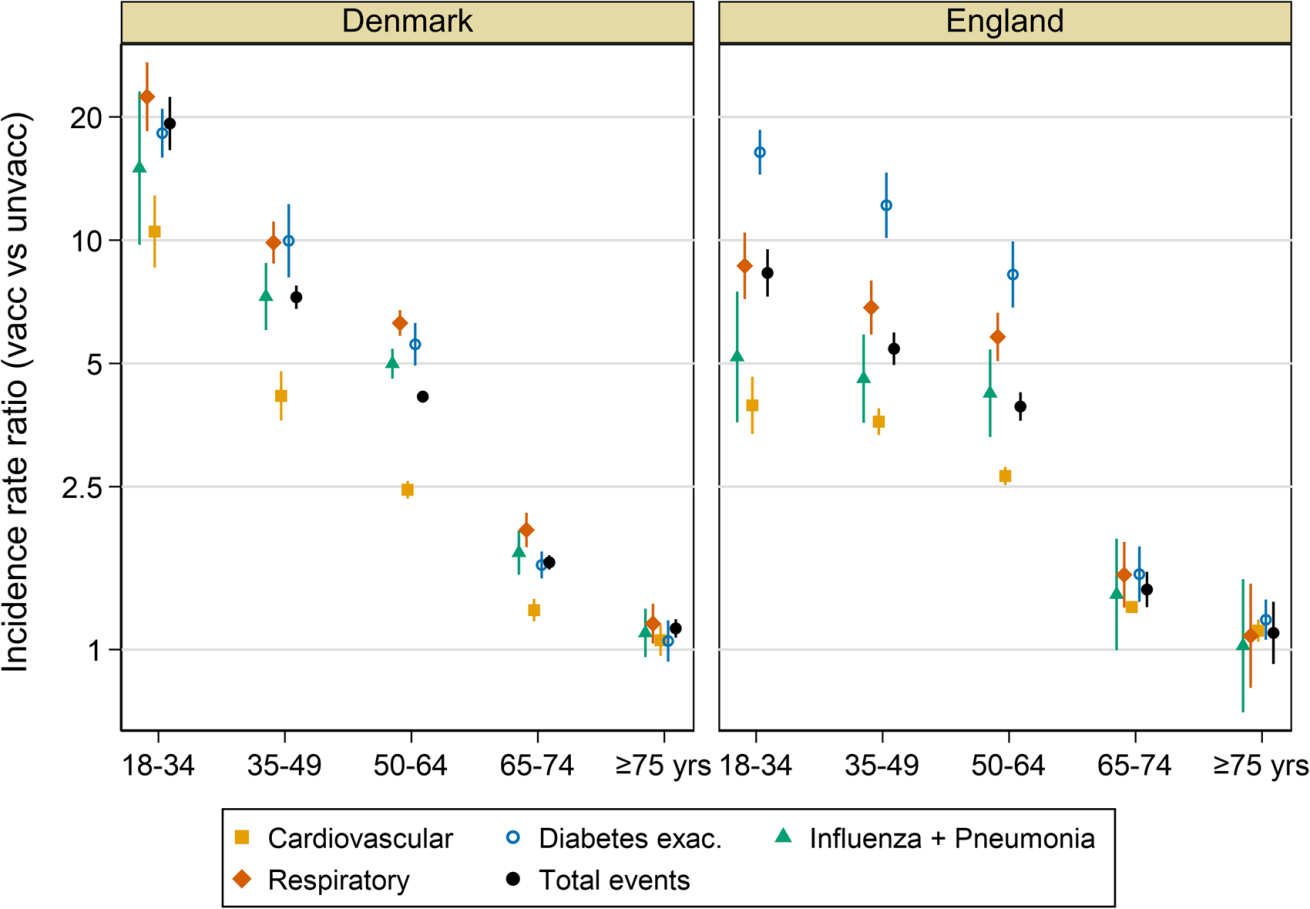
Incidence rate ratios (log scale) of average seasonal outcome rates in influenza vaccinated vs unvaccinated populations in Denmark and England, by age group. Error bars represent mean ± SEM. Underlying data in Supplementary Tables 4 and 5.

Outcome rates were higher in influenza vaccine recipients than non-recipients particularly in younger individuals in whom vaccination was less common, giving rise to IRRs between vaccine recipients and non-recipients which were nearly always >1 (Fig. 2 and Supplementary Tables 4 and 5). In Denmark, the IRR for all outcomes in the 18–34-year-old age group was 19.4 (95% CI: 18.1; 20.5) and declined in progressively older groups to 1.13 (1.12–1.14) in the ≥75 year old group. In England, the trend was similar with an IRR of 8.3 (95% CI: 8.0–8.7) in the youngest group, declining to 1.10 (95% CI: 1.08; 1.11) in the oldest. These trends were broadly similar across outcome groups with high IRRs in younger age groups declining to ~1 in those aged ≥75 years.

### Outcomes in individuals with existing high-risk conditions

**Fig. 3:**
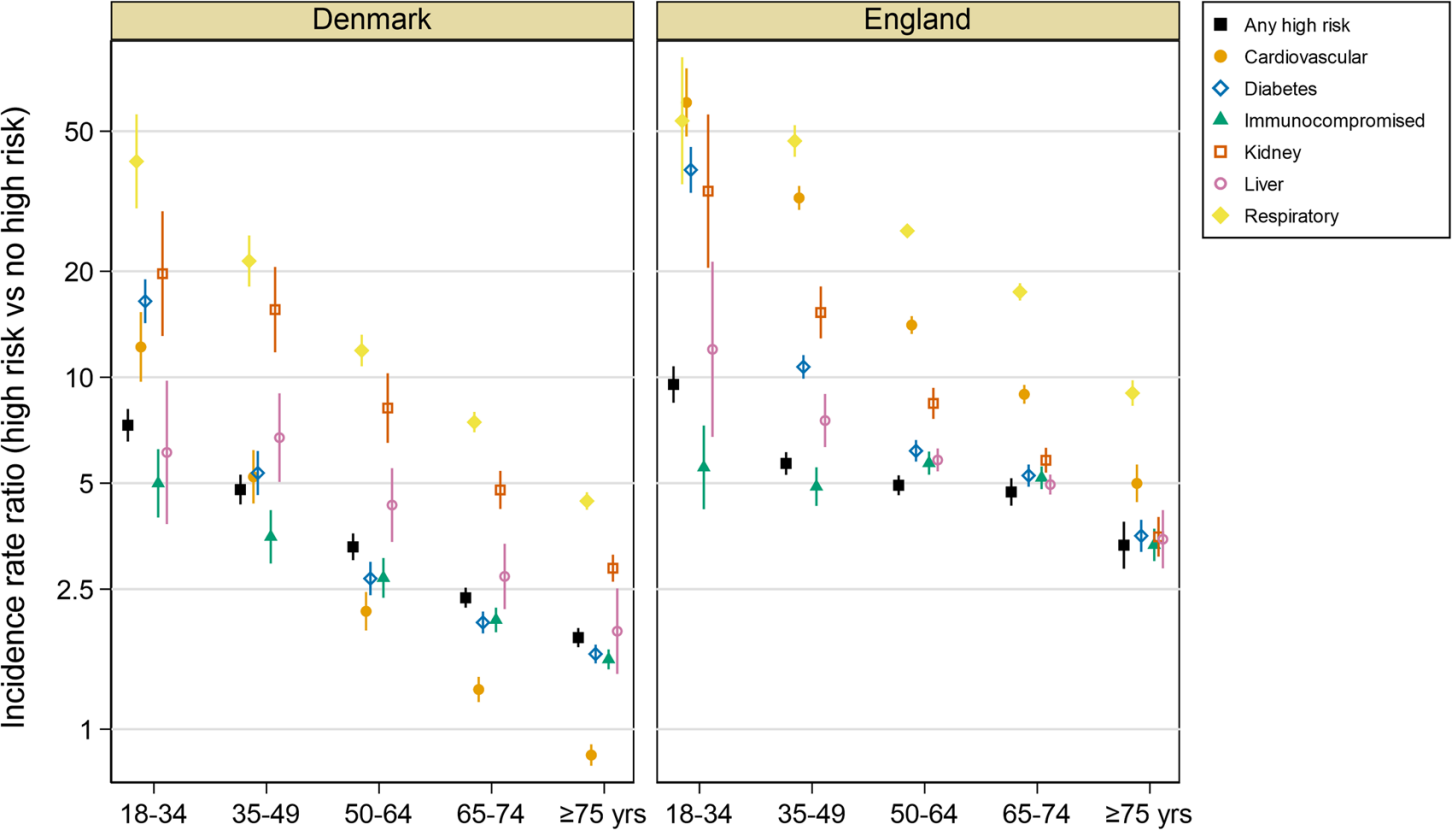
Incidence rate ratios (log scale) of total hospitalizations in individuals with any high-risk condition or specified high risk conditions vs those with none, by age group in Denmark and England. Error bars represent mean ± SEM.

Of the annual average of 46,984 hospitalizations captured in Denmark, 30,377 (65%) occurred in individuals with ≥1 high risk condition, an IR of 4014 (95% CI: 3998–4030) per 100,000, compared to 810 (807–813) in those without high risk conditions (Supplementary Table 6). Similarly, in England, most (81%) hospitalizations were in individuals with high risk conditions for an IR of 1271 (1266–1275) compared to 141 (140–142) in those without high-risk conditions (Supplementary Table 7). Among individual high risk groups, people with respiratory conditions experienced the highest rates of any hospitalization in both England and Denmark giving IRRs vs individuals with no high risk conditions of 17.5 (17.4–17.7) in Denmark and 46.3 (45.8–46.7) in England (Fig. 3, Supplementary Tables S8 and S9). IRRs in individuals with ≥1 high risk condition were highest in the 18–34-year-old age group (7.3 in Denmark and 9.5 in England), an effect driven by low hospitalization rates in healthy younger adults, and declined in older age groups. Significantly elevated incidence rates were observed in at-risk populations irrespective of their age.

### Sample size for a pragmatic RCT

**Fig. 4:**
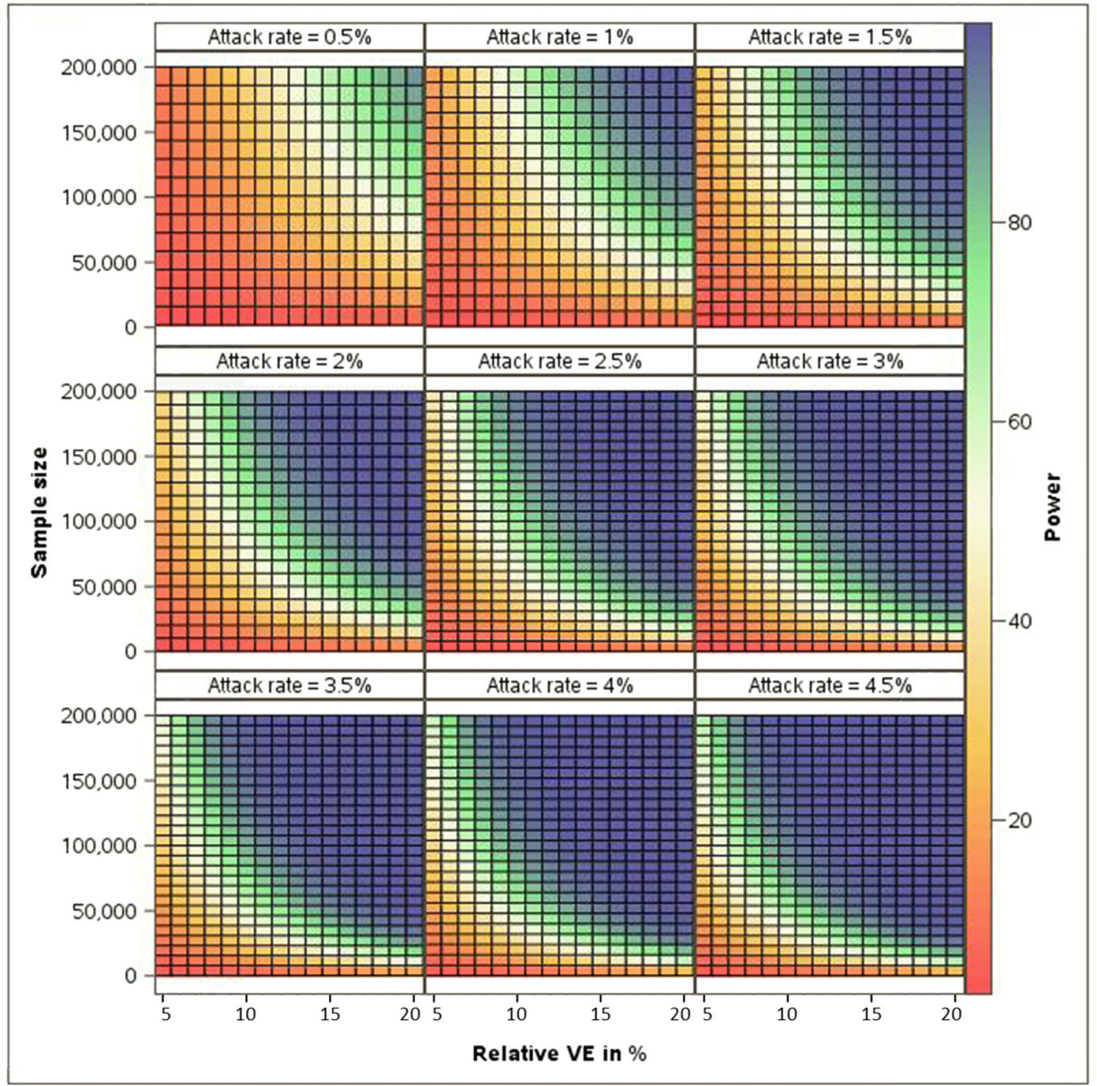
Power to demonstrate relative vaccine efficacy >0% under different rVE, attack rate and sample size assumptions. Power calculated by exact method, based on binomial distribution of cases in investigational group among overall number of cases. Attack rate representing the frequency of events is assumed in control group - Type I error 2.5% - 1:1allocation ratio. Selected attack rates reflect the range of incidence rates of events as estimated in our study (IR 1000/100,000 individuals = attack rate of 1%).

Under different rVE assumptions, a range of incidence rate scenarios (which we assumed as attack rates) representing the frequency of events reported above and a total sample size of 100,000, the power to conclude rVE >0 in a RCT varied from ~7% to ~100% (Fig. 4). With attack rates <0.5% or rVE <7%, power was low irrespective of other parameters. To achieve a power of 80% to ascertain a rVE of 10%, the frequency of events to be used as endpoints would need to be ≥1.5% in a population of at least 200,000 people. Rare event rates (<1%) as seen in certain populations would require even higher sample sizes for a similar expected rVE.

## Discussion

Our analysis over 8 years in Denmark and 9 years in England showed that ~1% of Danish and ~0.5% of English individuals were hospitalized for selected health events that could be associated with influenza every season, with rates varying significantly according to age and the presence of high-risk medical conditions. Among these events, respiratory hospitalizations were the most commonly seen in patients of all ages; the proportion of cardiovascular events increased markedly with age; diabetic exacerbations were exceedingly rare; and influenza as a primary diagnosis was reported in <2% of hospitalizations, a proportion which varied by season, synchronous with recorded epidemics^18^. Unsurprisingly, hospitalizations were more common in older adults: respiratory and cardiovascular hospitalizations were ~40 fold and ~100-fold higher in those aged ≥75 than in those aged 18–34 years.

The presence of high-risk medical conditions was strongly associated with hospitalization particularly in younger individuals but, even in older adults, high risk conditions were associated with a 2–3-fold elevated rate of hospitalization. Younger adults with cardiovascular or respiratory conditions experienced 10–50-times more hospitalizations than comparable individuals with no comorbidities, underlining the importance of chronic disease management in these vulnerable groups, irrespective of their age. The presence of high-risk conditions has been shown to elevate risk of severe and hospitalized influenza outcomes, these individuals benefit most from influenza vaccination, and could therefore be considered priorities for inclusion in influenza vaccine studies^19,20^. Hospitalization rates were up to 20-fold higher in younger influenza vaccine recipients compared with unvaccinated groups of the same age, most likely because vaccination is indicated only for high-risk groups in this age. Across all ages we observed <50% of high risk individuals received influenza vaccination annually, as is common in European countries^21^, and it is likely that only patients at highest risk, in frequent contact with health services for example, receive annual influenza vaccination. This confounding by indication or health care seeking bias—whereby baseline health condition rather than vaccination status predicts the frequency of healthcare events—has been well-described in the influenza VE literature^22,23^. The magnitude of disparity in event rates between vaccine recipients/non-recipients we observed highlights the challenges of confounder adjustment in observational VE/rVE studies, and therefore the need for randomized studies, to reliably measure the performance of influenza vaccines^24^.

This study was conducted to improve planning of rVE studies by identifying populations likely to suffer hospitalizations and therefore offer reduced sample sizes. For example, in Denmark the population with high-risk conditions experienced ~4x higher rate of outcomes than those without, corresponding to an improvement in power from ~30% to ~90%, with a sample of 50,000 if the true rVE is around 15%. To achieve the same power in the population without high-risk conditions would require >200,000 study participants and therefore incur significantly greater resources and may be unfeasible in many settings. Whether or not such a study is feasible would depend on the size of eligible population within a participating healthcare system, the ability to randomize that population into treatment groups and the frequency of the outcome of interest. Individually-randomized trials are more labor-intensive to conduct if a very large sample size is required to receive vaccination during a short period, which is the case for influenza vaccination campaigns that start shortly before the season^17^. There are wide variations in influenza season intensity, and studies may need to be conducted over a longer period if conducted in mild seasons.

Targeting populations at highest risk, in whom outcome rates were higher, would therefore improve efficiencies at the risk of reducing generalizability of study results, but because only high-risk and older adults are recommended for influenza vaccination in most countries, limiting inclusion may offer a feasible and relevant population for study^10^. Conversely, enrolling a highly comorbid population would result in a high background rate of non-specific events which are not vaccine-preventable, thereby ‘diluting’ and reducing rVE as endpoints become less specific, particularly in seasons with low influenza circulation where the proportion of attributable events would be low. This dilution effect may explain a recent study in patients with high-risk cardiovascular disease in which a high-dose inactivated influenza vaccine did not significantly reduce all-cause mortality or cardiopulmonary hospitalizations in comparison with a standard dose vaccine^25^. To increase specificity we conducted a thorough clinical validation of included codes and included only respiratory and cardiovascular hospitalizations which were considered likely to be associated with influenza based on assessment of previous clinical studies^3,5,8,9^. We assumed rVE scenarios of 5–20% based on existing data and identified a number of scenarios where pragmatic RCTs would provide high statistical power with a sample size of <200,000 participants (Fig. 4)^12,13,26,27^. However, the influenza-attributable burden of broader secondary outcomes remains incompletely understood and will vary over time: endpoint selection involves a compromise between frequency and specificity which affects rVE, and these assumptions will require refinement as additional evidence arises including from ongoing RCTs^17,28^.

This study was conducted in large databases capturing comprehensive healthcare outcomes with a long history of use for medical research, but databases are not perfect. VCRs are under-estimated because influenza vaccinations delivered at non-medical settings such as pharmacies or workplaces may not always be captured. Reassuringly, the VCR we captured from both Denmark and England are similar to those reported in routine national statistics^29,30^. Trends were consistent by country, though overall incidence rates were higher in Denmark, probably a result of differential healthcare investments or health systems specificities, healthcare seeking behavior or clinical thresholds for hospitalization^31^. These findings may not be generalizable to other healthcare settings or countries. Our study did not collect individual-level data so could not describe the effect modification of age on high-risk or vaccination status or intra-season correlations due to repeated observations of the same participants in multiple seasons. We included slightly different ICD-10 codes than some other researchers, differences which should be considered when interpreting the public health implications of a given rVE value^8,32^. Importantly, high-risk conditions in England were based on primary care consultations rather than the hospital contact data used in Denmark, likely explaining the higher prevalence of some conditions, notably asthma and kidney disorders, in England. Focusing on specificity, we captured only the primary/main reason for hospitalization and therefore may underestimate influenza: due to laboratory confirmation and coding practices, the full influenza burden in the US, for example, has been shown to be around 3-fold higher if codes relating to “any” rather than the primary diagnostic position are included^33,34^.

In conclusion, we identified groups at high risk of respiratory and cardiovascular events who would represent ideal populations for inclusion in pragmatic influenza vaccine controlled trials. In addition to older individuals, younger adults with high-risk conditions experienced frequent hospitalizations; enrolling this population in rVE studies would increase the probability of detecting true differences between influenza vaccine types and platforms, allowing policymakers to make informed decisions on vaccine recommendations for this priority population group. Such studies appear feasible, particularly if enrollment was limited to individuals aged >50 yrs and/or with high-risk conditions. Pragmatic RCTs such as these would represent a research tool to understand the influenza-attributable proportion of respiratory and non-respiratory diseases and the full public health benefits of influenza vaccines in different population age and risk groups.

## Methods

### Study design and population

We conducted a retrospective cohort study in the 2010/11–2017/18 influenza seasons from Denmark and the 2010/11–2018/19 seasons from England using large healthcare databases in each country. Populations aged ≥18 years on December 1st each year were included in seasonal cohorts and the number of hospitalized events occurring between December 1st and May 31st (defined as the influenza season) was divided by these denominators to calculate seasonal incidence rates of various outcomes stratified by age (18–34 years [yrs]; 35–49 yrs; 50–64 yrs; 65–74 yrs; ≥75 yrs), influenza vaccination status and the presence of clinical high risk (hereafter “high risk”) conditions. In both countries, influenza vaccination is recommended and provided free of charge for high-risk adults and adults aged ≥65 yrs^35,36^.

### Data sources

All Danish citizens are assigned a unique personal identification number which allows for exact linkage of nationwide administrative registers at the individual level. The Danish Civil Registration System, which records date of birth, emigration status and vital status for all persons residing in Denmark, was used to define cohorts^37^. The Danish National Patient Registry (DNPR) has shown high validity of cardiovascular diagnoses and captures all inpatient and outpatient hospital contacts coded in International Classification of Diseases 10 (ICD-10)^22,38^. The DNPR was used to count hospitalized events and to define high risk conditions. Influenza vaccination status was captured from the Danish National General Practitioners Reimbursement registry. Analyses were conducted by Danish researchers with access to raw, de-identified nationwide registry data in accordance with Danish law.

The UK Clinical Practice Research Datalink (CPRD) is a longitudinal and representative primary care database from a network of over 1,800 primary care practices and includes 16 million currently registered active patients^39–42^. This analysis used data from the CPRD GOLD and CPRD Aurum primary care databases to define vaccination and high risk status, linked to secondary care data from Hospital Episode Statistics Admitted Patient Care database to capture hospitalized outcomes rates of specified events^43^. Influenza vaccinations administered in GP practices or community pharmacies are captured in these electronic health records. Analysis of the CPRD data was conducted internally by CPRD researchers using databases of pseudonymized patient EHRs, therefore individual participant consent is not required. The study protocol was approved by the Independent Scientific Advisory Committee (ISAC) at the Medicines and Healthcare products Regulatory Agency (protocol ref 20_115 R0 A1).

### Outcome selection

We pre-specified groups of medical events, most of which were acute, based on previously documented and plausible associations with influenza, and which we considered outcomes of public health relevance for future pragmatic RCTs. ICD-10 coded primary discharge diagnoses (i.e., the main reason for hospitalization) resulting in hospitalization for ≥1 night were categorized into five groups: (1) influenza; (2) influenza and pneumonia; (3) respiratory; (4) cardiovascular; (5) exacerbations of diabetes. Groups were overlapping to explore the impact on incidence rate of including broader or more specific outcomes as potential study endpoints. The first occurrence of each event per season was included. The list of final codes within each category was selected from all “I” (cardiovascular), “J” (respiratory; of which J09-J11 were used to define ‘influenza’) and “E” (diabetic) ICD codes based on clinical review, available literature and discussion of the pathology and typical usage of those diagnostic codes in medical practice (Supplementary Table 1)^9,44,45^.

### Definition of high-risk conditions

Clinical high-risk conditions corresponding to eligibility for free annual influenza vaccination were modified from definitions used by the UK National Health Service and Danish Statens Serum Institute. They included cardiovascular disorders (including arrhythmias, congestive heart failure, ischemic heart disease and congenital heart disease), respiratory conditions (including asthma), hepatic and renal disorders, neurologic/neuromuscular disorders, blood disorders, metabolic/endocrine conditions including diabetes and conditions compromising the immune system^35,36^. For each condition, a list of ICD10 codes or prescription medication representing these diagnoses (for diabetes only) was defined (Supplementary Table 2). In the UK, primary care events coded with the SNOMED-CT architecture were mapped to these ICD-10 codes following review by a medical doctor (linked codes in Supplementary Data 1). Individuals diagnosed with qualifying events within the DNPR or CPRD primary care database within 3 years of the start of each influenza season, or a diabetes prescription <6 months before the start of each season, were included within that high-risk group for that season. Individuals receiving an influenza vaccination between August 1st and Jan 31st of the following year were considered vaccinated for that season.

### Statistical methods

The total number of incident outcome events experienced by the study population was summed for each season. Incidence rates of included outcomes, expressed as rates per 100,000 population, were calculated per season for populations overall and stratified by age group, high-risk condition, and influenza vaccination status. Average seasonal incidence rates over included seasons and their 95% confidence intervals (CIs) were estimated using a Poisson model with the number of events as the dependent variable, no independent variables, and the log of the population size as an offset, with Stata’s ‘glm’ command. In this parameterization, the exponential of the intercept is the incidence rate. The variance was adjusted by a scale factor equal to the deviance divided by the residual degrees of freedom to accounting for under/overdispersion in the underlying data^46,47^. Incidence rate ratios (IRR) and their 95% CIs comparing rates in: a) vaccinated vs unvaccinated and b) individuals with high-risk conditions vs those with no high-risk conditions, were similarly estimated with a Poisson model. A range of identified incidence rates were used to estimate the power of an rVE study by exact method, specifically coded in SAS, based on binomial distribution of cases in investigational groups among overall number of cases, a type I error of 2.5%, 1:1 allocation ratio and a maximum of 200,000 participants (100k per group). We assumed rVE ranging from 5–20% and expressed the result as a series of heatmaps. Analyses were conducted separately within the Danish and UK databases; subsequent manipulations were performed using Stata v 15.1 and SAS.

### Reporting summary

Further information on research design is available in the Nature Research Reporting Summary linked to this article.

## Supplementary information

Supplementary materials

Supplementary Data 1

Reporting summary

## Data Availability

These data were obtained from national EHR sources which are subject to local laws and regulations. UK data were provided by Clinical Practice Research Datalink (CPRD) under a licence from the UK Medicines and Healthcare products Regulatory Agency. CPRD data can be obtained by researchers following a successful application to CPRD. All Danish data are governed by the Danish Data Protection Agency and can only be made available to any additional researchers if a formal request is filed with the Danish Authorities.

## Code availability

No custom code or algorithms central to the conclusions were used.
