# Supplementary material for: The feasibility of pragmatic influenza vaccine randomized controlled real-world trials in Denmark and England
Source: NPJ Vaccines. 2022 Feb 23;7:25. doi: 10.1038/s41541-022-00444-6 (PMC8866398; doi:10.1038/s41541-022-00444-6)
Supplement: Supplementary file 1 — Supplementary materials [file 41541_2022_444_MOESM1_ESM.docx]

### **Supplementary table 1.** ICD-10 codes corresponding to included outcomes

| **Cardiovascular** | **Respiratory** | **Diabetic exacerbations** |
| --- | --- | --- |
| **CV: cardiac** | J09-J18: Influenza and pneumonia | E10.0: Type 1 diabetes mellitus with coma |
| I11: Hypertensive heart disease | J42: Unspecified chronic bronchitis | E11.0: Type 2 diabetes mellitus with coma |
| I13: Hypertensive heart and renal disease | J43: Emphysema | E12.0: Malnutrition-related diabetes mellitus with coma |
| I21: Myocardial infarction | J44: Other chronic obstructive pulmonary disease | E13.0: Other specified diabetes mellitus with coma |
| I22: Subsequent myocardial infarction | J45: Asthma | E14.0: Unspecified diabetes mellitus with coma |
| I25.5: Ischaemic cardiomyopathy | J96: Respiratory failure, not elsewhere classified | E10.1: Type 1 diabetes mellitus with ketoacidosis |
| I30: Acute pericarditis |  | E11.1 : Type 2 diabetes mellitus with ketoacidosis |
| I31: Other diseases of pericardium |  | E12.1: Malnutrition-related diabetes mellitus with ketoacidosis |
| I33: Acute and subacute endocarditis |  | E13.1: Other specified diabetes mellitus with ketoacidosis |
| I39: Endocarditis and heart valve disorders in diseases classified elsewhere |  | E14.1: Unspecified diabetes mellitus with ketoacidosis |
| I40: Acute myocarditis |  | E16.1: Other hypoglycaemia |
| I41: Myocarditis in diseases classified elsewhere |  | E16.2: Hypoglycaemia, unspecified |
| I42: Cardiomyopathy |  | R73: Abnormal glucose tolerance test |
| I46: Cardiac arrest |  |  |
| I50: Heart failure |  |  |
| **CV: vascular** |  |  |
| I60: Subarachnoid haemorrhage; |  |  |
| I61: Intracerebral haemorrhage; |  |  |
| I62: Other nontraumatic intracranial haemorrhage; |  |  |
| I63: Cerebral infarction |  |  |
| I64: Stroke, not specified as haemorrhage or infarction |  |  |
| I65: Occlusion and stenosis of precerebral arteries, not resulting in cerebral infarction; |  |  |
| I66: Occlusion and stenosis of cerebral arteries, not resulting in cerebral infarction |  |  |
| I67: Other cerebrovascular diseases |  |  |
| I68: Cerebrovascular disorders in diseases classified elsewhere; |  |  |
| I69: Sequelae of cerebrovascular disease |  |  |
| **CV: arrythmia** |  |  |
| I48: Atrial fibrillation and flutter |  |  |
| I49: Other cardiac arrhythmias |  |  |

### **Supplementary table 2.** ICD-10 codes and prescriptions applied to define high risk conditions

| **High risk condition** | **ICD-10 codes or prescriptions** |
| --- | --- |
| **Definition:** Codes recorded as Danish hospital encounters or UK primary care database within 3 years of the start of each influenza season. | |
| ***Diabetes*** | E10-E14  Prescription for glucose lowering medication (ATC A10) 6 months prior to inclusion (season start) |
| ***Endocrine disorders*** | E03 - Other hypothyroidism E04 - Other nontoxic goitre E05 - Thyrotoxicosis [hyperthyroidism] E06 - Thyroiditis E27 - Other disorders of adrenal gland |
| ***Respiratory*** | J43 - emphysema J44 - COPD) J47 - bronchiectasis E84 - cystic fibrosis J81 - Pulmonary oedema J84 - Interstitial lung disease J85 - Abscess of lung and mediastinum A15, A16 - Respiratory tuberculosis Z94.2 - Lung transplant status  D86.0 - Sarcoidosis of lung |
| ***Asthma*** | J45 - Asthma |
| ***Liver disorders*** | K70 - Alcoholic liver disease K713, K714, K715, K716, K717 - chronic toxic liver conditions K721, K729 - chronic hep failure K73 - chronic hepatitis K74 - cirrosis K766, K769 - portal hypertension B18-B19 - chronic and unspecified viral hepatitis |
| ***Kidney*** | N18-N19 - chronic kidney disease Z49 - Care involving dialysis Z99.2 - Dependence on renal dialysis I12 - Hypertensive renal disease |
| ***Neurological/neuromuscular*** | G20 - parkinsons disease G35 - MS G710 - muscular dystrophy G30, F00 - Alzheimers F01-F02 - Dementia G122 - ALS |
| ***Blood disorders*** | D55-D59 - haemolytic anemias D63 - Anemia in chronic disease |
| ***Cardiovascular*** | I20-I25 - ischemic heart disease I50 - heart failure I42-I43 - cardiomyopathy I34-I37 - valvular disease I48 - atrial fibrillaton I60-I69 - cerebrovascular disease I70, I74 - peripheral vascular disease I27-I28 - pulm circulation disease Q251 - coarctatio of the aorta Q201 - Double outlet right ventricle  Q203 - d-transposition  Q225 - Ebstein anomaly Q234 - hypoplastic left heart Q234 - hypoplastic left heart Q205 - single ventricle Q262 - TAPV Q231 - tetralogy of fallot Q224 - tetralogy of fallot Q200 - truncus arteriosus |
| ***Immunocompromised*** | B20-B24 - HIV C00-C97 - cancer L93 - lupus M05-M06 - RA  Z940-Z944, Z948A - Solid organ transplant  K50-K51 - inflammatory bowel disease |

### **Supplementary Data 1** SNOMED CT codes used in the UK for primary diagnosis and their linkage to ICD-10 codes used to define high risk conditions. [attached Excel file]

### **Supplementary figure 1.** Influenza vaccine coverage rate among overall population and those with and without high risk conditions by age groups in Denmark (2010/11 – 2017/18) and England (from 2010/11 – 2018/19). Data are averages of included seasons.


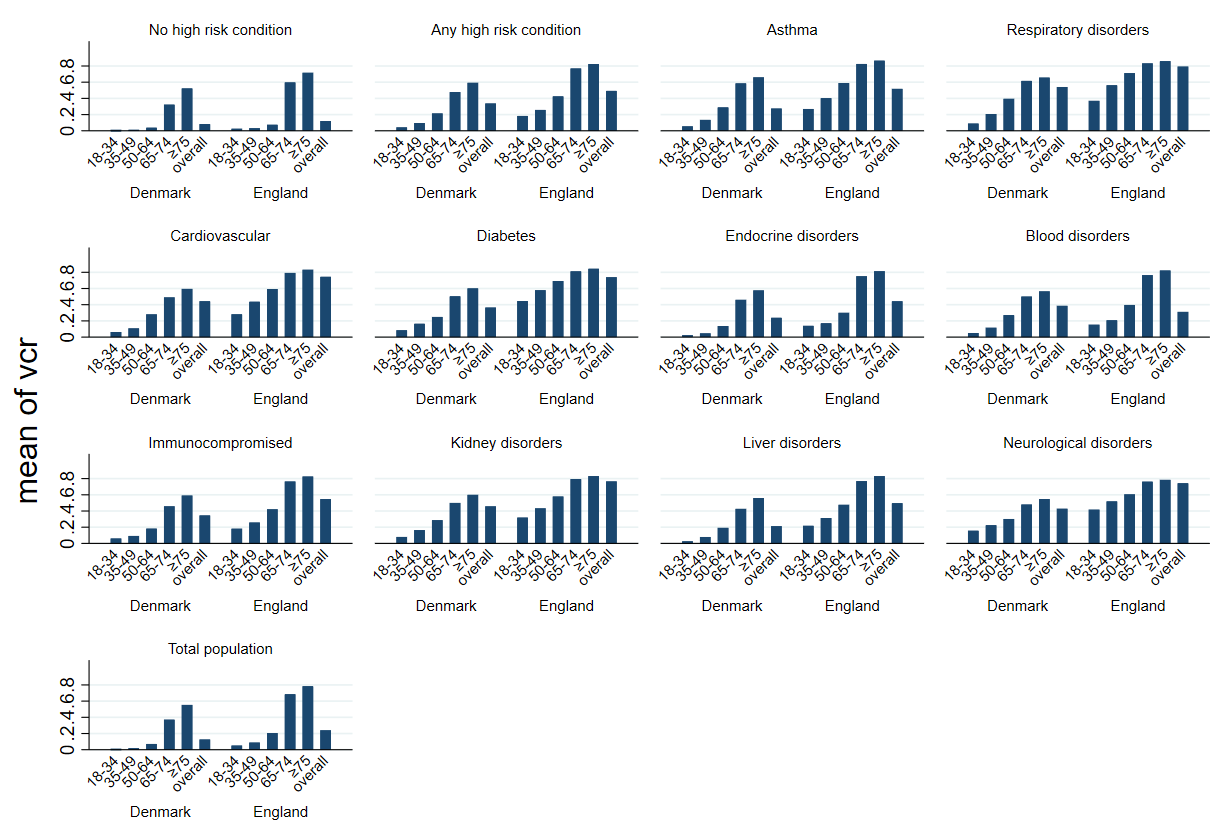


### **Supplementary table 3.** Seasonal average influenza vaccine coverage rate in Denmark and England, by risk status

| **Age group:** | **18-34 years** | **35-49 years** | **50-64 years** | **65-74 years** | **≥75 years** | **Total population** |
| --- | --- | --- | --- | --- | --- | --- |
| **Denmark** | | | | | | |
| No high risk condition | 0% | 1% | 4% | 32% | 52% | 8% |
| Any high risk condition | 4% | 10% | 21% | 48% | 59% | 34% |
| Asthma | 5% | 13% | 29% | 59% | 66% | 27% |
| Respiratory disorders | 9% | 21% | 39% | 62% | 66% | 54% |
| Cardiovascular | 6% | 11% | 28% | 49% | 60% | 44% |
| Diabetes | 8% | 16% | 25% | 50% | 60% | 36% |
| Endocrine disorders | 2% | 5% | 14% | 46% | 58% | 24% |
| Blood disorders | 5% | 12% | 27% | 50% | 56% | 39% |
| Immunocompromised | 6% | 9% | 18% | 46% | 59% | 35% |
| Kidney disorders | 8% | 16% | 29% | 50% | 60% | 46% |
| Liver disorders | 3% | 8% | 19% | 43% | 56% | 21% |
| Neurological disorders | 16% | 22% | 30% | 48% | 55% | 43% |
| Total population | 1% | 2% | 7% | 37% | 55% | 13% |
| **England** | | | | | | |
| No high risk condition | 3% | 3% | 7% | 60% | 72% | 12% |
| Any high risk condition | 18% | 26% | 43% | 77% | 82% | 49% |
| Asthma | 27% | 40% | 59% | 83% | 87% | 52% |
| Respiratory disorders | 37% | 56% | 71% | 83% | 86% | 79% |
| Cardiovascular | 28% | 44% | 59% | 79% | 83% | 74% |
| Diabetes | 44% | 58% | 69% | 81% | 84% | 74% |
| Endocrine disorders | 14% | 17% | 30% | 75% | 81% | 44% |
| Blood disorders | 15% | 21% | 40% | 76% | 82% | 31% |
| Immunocompromised | 18% | 26% | 42% | 76% | 83% | 55% |
| Kidney disorders | 32% | 43% | 58% | 79% | 83% | 77% |
| Liver disorders | 22% | 31% | 48% | 77% | 83% | 50% |
| Neurological disorders | 42% | 52% | 61% | 76% | 78% | 74% |
| Total population | 5% | 9% | 21% | 69% | 78% | 24% |

### **Supplementary table 4.** Seasonal average number (#) and incidence rates (IR) of in-hospital selected outcomes per 100,000 population by age group by influenza vaccination status, and their incidence rate ratios, Denmark (from 2010/11 – 2017/18).

| **Age group:** |  | **18-34 years** | **35-49 years** | **50-64 years** | **65-74 years** | **≥75 years** | **All ages** |
| --- | --- | --- | --- | --- | --- | --- | --- |
| **Influenza vaccinated population** | | | | | | | |
| **Any hospitalization** | # | 137 | 409 | 2324 | 6071 | 11723 | 20663 |
|  | IR | 2085 (1941; 2240) | 1870 (1785; 1958) | 3001 (2940; 3063) | 2694 (2627; 2763) | 5026 (4876; 5180) | 3661 (3564; 3760) |
| **Respiratory hospitalizations** | # | 107 | 277 | 1489 | 3446 | 6906 | 12224 |
|  | IR | 1622 (1525; 1725) | 1265 (1197; 1338) | 1923 (1864; 1983) | 1529 (1446; 1617) | 2961 (2773; 3161) | 2165 (2047; 2290) |
| Influenza + pneumonia | # | 48 | 139 | 748 | 1926 | 4661 | 7522 |
|  | IR | 723 (642; 814) | 636 (581; 697) | 965 (933; 999) | 855 (800; 914) | 1998 (1848; 2161) | 1333 (1244; 1427) |
| Influenza | # | 3 | 11 | 35 | 70 | 132 | 251 |
|  | IR | 47 (27; 82) | 49 (29; 82) | 45 (24; 85) | 31 (16; 62) | 57 (25; 126) | 44 (22; 91) |
| **Cardiovascular hospitalizations** | # | 11 | 81 | 539 | 1692 | 3569 | 5891 |
|  | IR | 171 (135; 216) | 370 (332; 413) | 695 (660; 732) | 751 (714; 789) | 1530 (1457; 1607) | 1044 (994; 1096) |
| **Diabetic hospitalizations** | # | 14 | 20 | 46 | 75 | 113 | 268 |
|  | IR | 216 (190; 246) | 93 (81; 105) | 60 (53; 67) | 33 (31; 35) | 48 (44; 53) | 48 (45; 51) |
| **Influenza unvaccinated population** | | | | | | | |
| **Any hospitalization** | # | 1276 | 2941 | 7335 | 6314 | 8454 | 26320 |
|  | IR | 108 (102; 114) | 258 (251; 265) | 723 (709; 737) | 1650 (1601; 1700) | 4461 (4307; 4621) | 674 (647; 701) |
| **Respiratory hospitalizations** | # | 854 | 1464 | 3107 | 2986 | 4849 | 13260 |
|  | IR | 72 (67; 78) | 128 (121; 136) | 306 (291; 323) | 780 (727; 838) | 2559 (2377; 2754) | 339 (314; 367) |
| Influenza + pneumonia | # | 569 | 997 | 1961 | 1898 | 3448 | 8873 |
|  | IR | 48 (42; 56) | 87 (81; 94) | 193 (183; 205) | 496 (453; 543) | 1819 (1662; 1992) | 227 (207; 249) |
| Influenza | # | 94 | 108 | 129 | 95 | 128 | 553 |
|  | IR | 8 (5.4; 12) | 9.4 (6.8; 13) | 13 (8; 20) | 25 (13; 47) | 68 (31; 149) | 14 (9; 23) |
| **Cardiovascular hospitalizations** | # | 192 | 1014 | 2871 | 2302 | 2751 | 9130 |
|  | IR | 16 (16; 17) | 89 (85; 92) | 283 (279; 288) | 601 (583; 620) | 1452 (1370; 1539) | 234 (230; 237) |
| **Diabetic hospitalizations** | # | 140 | 106 | 109 | 79 | 87 | 521 |
|  | IR | 12 (11; 12) | 9.3 (8.4; 10) | 11 (10; 11) | 21 (20; 22) | 46 (43; 49) | 13 (13; 14) |
| **Incidence rate ratio (vaccinated vs unvaccinated)** | | | | | | | |
| **Any hospitalization** |  | 19.3 (16.6; 22.4) | 7.3 (6.8; 7.7) | 4.1 (4; 4.3) | 1.6 (1.6; 1.7) | 1.1 (1.1; 1.2) | 5.4 (5.2; 5.7) |
| **Respiratory hospitalizations** |  | 22.4 (18.5; 27.2) | 9.9 (8.8; 11.1) | 6.3 (5.8; 6.7) | 2 (1.8; 2.2) | 1.2 (1; 1.3) | 6.4 (5.7; 7.1) |
| Influenza + pneumonia |  | 15 (9.7; 23.1) | 7.3 (6; 8.8) | 5 (4.6; 5.4) | 1.7 (1.5; 1.9) | 1.1 (1; 1.3) | 5.9 (5.2; 6.7) |
| Influenza |  | 6 (1; 36.8) | 5.2 (2; 13.5) | 3.6 (1.4; 9.4) | 1.3 (0.4; 3.7) | 0.8 (0.2; 3.1) | 3.1 (1.2; 8.5) |
| **Cardiovascular hospitalizations** |  | 10.5 (8.6; 12.9) | 4.2 (3.6; 4.8) | 2.5 (2.3; 2.6) | 1.2 (1.2; 1.3) | 1.1 (1; 1.2) | 4.5 (4.3; 4.7) |
| **Diabetic hospitalizations** |  | 18.3 (15.9; 20.9) | 10 (8.1; 12.2) | 5.6 (4.9; 6.3) | 1.6 (1.5; 1.7) | 1 (0.9; 1.2) | 3.6 (3.3; 3.8) |

Data are averages of included seasons and 95% confidence intervals (in brackets) from the Poisson distribution.

### **Supplementary table 5.** Seasonal average number (#) and incidence rates (IR) of in-hospital selected outcomes per 100,000 population by age group by influenza vaccination status, England (from 2010/11 – 2018/19).

| **Age group:** |  | **18-34 years** | **35-49 years** | **50-64 years** | **65-74 years** | **≥75 years** | **All ages** |
| --- | --- | --- | --- | --- | --- | --- | --- |
| **Influenza vaccinated population** | | | | | | | |
| **Any hospitalization** | # | 320 | 820 | 3343 | 6191 | 14318 | 24992 |
|  | IR | 294 (282; 307) | 487 (474; 500) | 994 (952; 1037) | 1113 (1059; 1170) | 2595 (2398; 2808) | 1452 (1372; 1536) |
| **Respiratory hospitalizations** | # | 224 | 507 | 1935 | 3385 | 7590 | 13642 |
|  | IR | 206 (194; 219) | 301 (290; 312) | 575 (545; 607) | 609 (555; 667) | 1376 (1197; 1580) | 792 (717; 876) |
| Influenza + pneumonia | # | 76 | 214 | 843 | 1764 | 5239 | 8136 |
|  | IR | 70 (61; 80) | 127 (115; 140) | 251 (219; 288) | 317 (269; 374) | 950 (789; 1142) | 473 (402; 556) |
| Influenza | # | 12 | 23 | 66 | 99 | 185 | 384 |
|  | IR | 11 (5; 22) | 14 (7; 26) | 20 (10; 40) | 18 (8; 39) | 33 (13; 83) | 22 (10; 50) |
| **Cardiovascular hospitalizations** | # | 35 | 264 | 1368 | 2848 | 6974 | 11489 |
|  | IR | 33 (31; 35) | 157 (150; 164) | 407 (393; 421) | 512 (504; 521) | 1264 (1236; 1292) | 667 (656; 679) |
| **Diabetic hospitalizations** | # | 62 | 60 | 101 | 96 | 234 | 552 |
|  | IR | 57 (51; 64) | 35 (32; 39) | 30 (27; 33) | 17 (16; 18) | 42 (41; 44) | 32 (31; 33) |
| **Influenza unvaccinated population** | | | | | | | |
| **Any hospitalization** | # | 713 | 1581 | 3304 | 2024 | 3586 | 11208 |
|  | IR | 35 (32; 39) | 90 (84; 95) | 253 (240; 267) | 794 (742; 850) | 2363 (2116; 2638) | 204 (188; 222) |
| **Respiratory hospitalizations** | # | 480 | 775 | 1292 | 1019 | 1933 | 5499 |
|  | IR | 24 (21; 27) | 44 (39; 49) | 99 (88; 111) | 400 (356; 449) | 1273 (1079; 1502) | 100 (88; 114) |
| Influenza + pneumonia | # | 272 | 488 | 774 | 593 | 1410 | 3536 |
|  | IR | 13 (11; 17) | 28 (24; 32) | 59 (50; 70) | 233 (194; 279) | 929 (762; 1132) | 64 (54; 77) |
| Influenza | # | 55 | 67 | 85 | 40 | 57 | 303 |
|  | IR | 2.7 (1.5; 5.0) | 3.8 (2; 7) | 6.5 (3.3; 13) | 16 (7; 36) | 37 (15; 91) | 5.5 (2.8; 11) |
| **Cardiovascular hospitalizations** | # | 166 | 768 | 1999 | 1027 | 5684 | 1725 |
|  | IR | 8.2 (7.6; 8.9) | 44 (42; 45) | 153 (149; 158) | 403 (390; 417) | 1136 (1073; 1203) | 104 (99; 108) |
| **Diabetic hospitalizations** | # | 70 | 51 | 48 | 29 | 54 | 252 |
|  | IR | 3.5 (3.3; 3.7) | 2.9 (2.5; 3.4) | 3.6 (3.3; 4.1) | 11 (10; 13) | 36 (32; 40) | 4.6 (4.3; 4.9) |
| **Incidence rate ratio (vaccinated vs unvaccinated)** | | | | | | | |
| **Any hospitalization** |  | 8.3 (7.3; 9.5) | 5.4 (5; 6) | 3.9 (3.6; 4.3) | 1.4 (1.3; 1.5) | 1.1 (0.9; 1.3) | 7.1 (6.3; 8) |
| **Respiratory hospitalizations** |  | 8.7 (7.2; 10.4) | 6.8 (5.9; 8) | 5.8 (5.1; 6.7) | 1.5 (1.3; 1.8) | 1.1 (0.8; 1.4) | 7.9 (6.5; 9.7) |
| Influenza + pneumonia |  | 5.2 (3.6; 7.5) | 4.6 (3.6; 5.9) | 4.2 (3.3; 5.4) | 1.4 (1; 1.9) | 1 (0.7; 1.5) | 7.3 (5.4; 9.9) |
| Influenza |  | 4 (1.1; 15.4) | 3.6 (1.1; 11.7) | 3 (1; 9.5) | 1.1 (0.3; 4.6) | 0.9 (0.2; 5.1) | 4 (1.2; 13.7) |
| **Cardiovascular hospitalizations** |  | 4 (3.4; 4.6) | 3.6 (3.3; 3.9) | 2.7 (2.5; 2.8) | 1.3 (1.2; 1.3) | 1.1 (1; 1.2) | 6.4 (6.1; 6.8) |
| **Diabetic hospitalizations** |  | 16.4 (14.5; 18.6) | 12.2 (10.1; 14.6) | 8.2 (6.8; 9.9) | 1.5 (1.3; 1.8) | 1.2 (1.1; 1.3) | 7 (6.4; 7.7) |

Data are averages of included seasons and 95% confidence intervals (in brackets) from the Poisson distribution.

### **Supplementary table 6.** Number (#) of any hospitalizations (including cardiovascular, respiratory or diabetic) and their incidence rates per 100,000 individuals (IR) in individuals with no existing high risk conditions, any high risk conditions and individual high risk conditions and corresponding incidence rate ratios from Denmark (from 2010 – 2017). Data are averages of included seasons and 95% confidence intervals (in brackets) from the Poisson distribution.

| **Age group:** |  | **18-34 years** | **35-49 years** | **50-64 years** | **65-74 years** | **≥75 years** | **Total population** | |
| --- | --- | --- | --- | --- | --- | --- | --- | --- |
| No high risk condition | # | 1333 | 2988 | 7671 | 7989 | 10087 | 30069 | |
|  | IR | 119 (112-127) | 285 (267-304) | 863 (814-915) | 1908 (1830-1989) | 4195 (4043-4352) | 810 (773-849) | |
| Any high risk condition | # | 594 | 1566 | 5769 | 8531 | 13917 | 30377 | |
|  | IR | 872 (804-946) | 1367 (1279-1461) | 2847 (2668-3038) | 4509 (4289-4741) | 7635 (7276-8012) | 4014 (3820-4218) | |
| Asthma | # | 180 | 243 | 406 | 389 | 494 | 1712 | |
|  | IR | 1909 (1296-2812) | 2360 (2203-2527) | 4059 (3819-4314) | 6546 (6048-7085) | 11825 (11183-12504) | 4297 (4077-4529) | |
| Respiratory disorders | # | 64 | 264 | 1642 | 2874 | 4595 | 9439 | |
|  | IR | 4902 (3602-6670) | 6098 (5723-6498) | 10279 (9860-10717) | 14222 (13636-14832) | 18667 (17956-19407) | 14209 (13666-14773) | |
| Cardiovascular | # | 58 | 253 | 1090 | 1786 | 3366 | 6553 | |
|  | IR | 1456 (1362-1556) | 1486 (1365-1618) | 1867 (1757-1984) | 2474 (2334-2623) | 3542 (3348-3748) | 2657 (2527-2795) | |
| Diabetes | # | 197 | 407 | 1612 | 2594 | 3554 | 8363 | |
|  | IR | 1963 (1793-2150) | 1524 (1411-1645) | 2313 (2180-2454) | 3837 (3652-4031) | 6862 (6585-7150) | 3704 (3522-3895) | |
| Endocrine disorders | # | 26 | 84 | 293 | 448 | 820 | 1670 | |
|  | IR | 352 (299-414) | 597 (547-651) | 1774 (1669-1886) | 4145 (3932-4369) | 7718 (7252-8213) | 2812 (2663-2969) | |
| Blood disorders | # | 7 | 12 | 51 | 92 | 185 | 346 | |
|  | IR | 1366 (1009-1849) | 2408 (1793-3235) | 7075 (6419-7799) | 9681 (9057-10348) | 12526 (11973-13106) | 8422 (7903-8976) | |
| Immunocompromised | # | 68 | 235 | 1092 | 1995 | 3023 | 6413 | |
|  | IR | 596 (529-672) | 1004 (945-1065) | 2323 (2156-2502) | 3898 (3673-4137) | 6624 (6336-6926) | 3590 (3410-3780) | |
| Kidney disorders | # | 20 | 85 | 285 | 529 | 1144 | 2063 | |
|  | IR | 2352 (1872-2956) | 4438 (4128-4771) | 7054 (6462-7699) | 9126 (8641-9639) | 12024 (11492-12581) | 9330 (8930-9748) | |
| Liver disorders | # | 14 | 82 | 244 | 158 | 94 | 591 | |
|  | IR | 730 (609-875) | 1921 (1720-2145) | 3740 (3476-4025) | 5184 (4942-5437) | 7966 (7247-8757) | 3504 (3311-3709) | |
| Neurological disorders | # | 46 | 76 | 185 | 305 | 891 | 1502 | |
|  | IR | 3071 (2884-3270) | 2031 (1774-2325) | 3387 (3194-3591) | 5300 (5060-5550) | 6798 (6454-7160) | 5085 (4875-5304) | |
| Total population | # | 1414 | 3350 | 9659 | 12385 | 20177 | 46984 | |
|  | IR | 119 (113-126) | 288 (282-295) | 885 (872-898) | 2037 (1995-2080) | 4773 (4630-4920) | 1051 (1022-1081) | |
| **Incidence rate ratio (vs no high-risk condition)** | | | | | | | |  |
| Any high risk condition |  | 7.3 (6.6; 8.1) | 4.8 (4.4; 5.3) | 3.3 (3; 3.6) | 2.4 (2.2; 2.5) | 1.8 (1.7; 1.9) | 5 (4.6; 5.3) | |
| Asthma |  | 16 (11.6; 22) | 8.3 (7; 9.8) | 4.7 (3.9; 5.7) | 3.4 (3; 4) | 2.8 (2.5; 3.2) | 5.3 (4.6; 6.1) | |
| Respiratory disorders |  | 41 (30.2; 55.8) | 21.4 (18.1; 25.3) | 11.9 (10.7; 13.2) | 7.5 (7; 8) | 4.5 (4.2; 4.7) | 17.5 (16.3; 18.9) | |
| Cardiovascular |  | 12.2 (9.7; 15.3) | 5.2 (4.4; 6.2) | 2.2 (1.9; 2.5) | 1.3 (1.2; 1.4) | 0.8 (0.8; 0.9) | 3.3 (3; 3.6) | |
| Diabetes |  | 16.4 (14.2; 19) | 5.3 (4.6; 6.2) | 2.7 (2.4; 3) | 2 (1.9; 2.2) | 1.6 (1.5; 1.7) | 4.6 (4.2; 5) | |
| Endocrine disorders |  | 2.9 (2.1; 4.2) | 2.1 (1.6; 2.8) | 2.1 (1.6; 2.6) | 2.2 (1.9; 2.5) | 1.8 (1.7; 2) | 3.5 (3; 4) | |
| Blood disorders |  | 11.4 (5.8; 22.6) | 8.4 (4; 18) | 8.2 (4.9; 13.7) | 5.1 (3.8; 6.7) | 3 (2.5; 3.6) | 10.4 (7.6; 14.2) | |
| Immunocompromised |  | 5 (4; 6.2) | 3.5 (3; 4.2) | 2.7 (2.4; 3.1) | 2 (1.9; 2.2) | 1.6 (1.5; 1.7) | 4.4 (4.1; 4.8) | |
| Kidney disorders |  | 19.7 (13.1; 29.6) | 15.6 (11.8; 20.6) | 8.2 (6.5; 10.3) | 4.8 (4.2; 5.4) | 2.9 (2.6; 3.1) | 11.5 (10.1; 13.2) | |
| Liver disorders | | 6.1 (5.1; 7.4) | 6.1 (3.8; 9.8) | 6.7 (5; 9) | 4.3 (3.4; 5.5) | 2.7 (2.2; 3.4) | 1.9 (1.4; 2.5) | |
| Neurological disorders |  | 25.7 (20; 33.1) | 7.1 (5.2; 9.7) | 3.9 (3; 5.2) | 2.8 (2.4; 3.2) | 1.6 (1.5; 1.8) | 6.3 (5.4; 7.3) | |
| Total population |  | 1 (0.9; 1.1) | 1 (0.9; 1.1) | 1 (1; 1.1) | 1.1 (1; 1.1) | 1.1 (1.1; 1.2) | 1.3 (1.2; 1.4) | |

### **Supplementary table 7.** Number (#) of any hospitalizations (including cardiovascular, respiratory or diabetic) and their incidence rates per 100,000 individuals (IR) in individuals with no existing high risk conditions, any high risk conditions and individual high risk conditions and corresponding incidence rate ratios from England (from 2010/11 – 2018/19). Data are averages of included seasons and 95% confidence intervals (in brackets) from the Poisson distribution.

| **Age group:** |  | **18-34 years** | **35-49 years** | **50-64 years** | **65-74 years** | **≥75 years** | **Total population** |
| --- | --- | --- | --- | --- | --- | --- | --- |
| No high risk condition | # | 356 | 832 | 1699 | 1382 | 2667 | 6936 |
|  | IR | 20 (18; 23) | 57 (54; 60) | 165 (160; 169) | 349 (342; 356) | 1029 (981; 1079) | 141 (137; 146) |
| Any high risk condition | # | 678 | 1569 | 4948 | 6833 | 15237 | 29264 |
|  | IR | 192 (182; 202) | 326 (310; 343) | 812 (777; 848) | 1647 (1565; 1734) | 3429 (3161; 3721) | 1271 (1188; 1358) |
| Asthma | # | 426 | 756 | 1571 | 1644 | 2934 | 7331 |
|  | IR | 285 (263; 308) | 497 (468; 527) | 1140 (1094; 1188) | 2238 (2140; 2341) | 4764 (4436; 5117) | 1276 (1195; 1362) |
| Respiratory disorders | # | 18 | 206 | 1615 | 2957 | 4881 | 9677 |
|  | IR | 1075 (814; 1420) | 2690 (2478; 2921) | 4290 (4155; 4430) | 6101 (5844; 6369) | 9276 (8732; 9854) | 6535 (6213; 6873) |
| Cardiovascular | # | 58 | 339 | 1734 | 3036 | 8651 | 13820 |
|  | IR | 1212 (1121; 1311) | 1853 (1766; 1944) | 2317 (2200; 2440) | 3122 (2979; 3271) | 5138 (4743; 5566) | 3801 (3557; 4062) |
| Diabetes | # | 152 | 349 | 1396 | 2138 | 4431 | 8466 |
|  | IR | 780 (754; 806) | 614 (586; 643) | 1017 (951; 1088) | 1834 (1724; 1952) | 3645 (3355; 3961) | 1874 (1745; 2013) |
| Endocrine disorders | # | 26 | 86 | 292 | 424 | 1134 | 1961 |
|  | IR | 139 (116; 166) | 223 (208; 239) | 559 (530; 590) | 1275 (1200; 1354) | 3163 (2939; 3405) | 1100 (1047; 1156) |
| Blood disorders | # | 5 | 6 | 14 | 13 | 39 | 77 |
|  | IR | 130 (107; 158) | 214 (167; 273) | 880 (774; 1000) | 1869 (1478; 2363) | 4958 (4299; 5719) | 803 (708; 912) |
| Immunocompromised | # | 36 | 104 | 509 | 962 | 2149 | 3761 |
|  | IR | 111 (102; 122) | 280 (257; 306) | 939 (866; 1017) | 1808 (1675; 1951) | 3436 (3116; 3788) | 1569 (1462; 1683) |
| Kidney disorders | # | 12 | 61 | 362 | 934 | 3633 | 5001 |
|  | IR | 679 (499; 923) | 876 (765; 1003) | 1388 (1234; 1560) | 2027 (1862; 2207) | 3622 (3238; 4052) | 2761 (2479; 3076) |
| Liver disorders | # | 8 | 46 | 160 | 133 | 118 | 465 |
|  | IR | 241 (194; 300) | 432 (390; 478) | 958 (910; 1009) | 1729 (1629; 1836) | 3565 (3114; 4083) | 1119 (1053; 1190) |
| Neurological disorders | # | 7 | 16 | 60 | 235 | 1741 | 2059 |
|  | IR | 517 (430; 622) | 396 (335; 469) | 830 (743; 929) | 2070 (1839; 2329) | 3349 (2889; 3881) | 2714 (2343; 3143) |
| Total population | # | 1033 | 2401 | 6647 | 8215 | 17904 | 36200 |
|  | IR | 49 (45; 52) | 124 (118; 131) | 405 (386; 425) | 1013 (964; 1065) | 2545 (2337; 2771) | 502 (467; 539) |
| **Incidence rate ratios, vs ‘no high risk condition’ group** | | | | | | | |
| Any high risk condition |  | 9.5 (8.5; 10.7) | 5.7 (5.3; 6.1) | 4.9 (4.6; 5.3) | 4.7 (4.3; 5.2) | 3.3 (2.9; 3.9) | 9 (8.1; 10) |
| Asthma |  | 14.2 (12.4; 16.3) | 8.7 (8; 9.4) | 6.9 (6.6; 7.3) | 6.4 (6.1; 6.7) | 4.6 (4.2; 5.1) | 9 (8.4; 9.7) |
| Respiratory disorders |  | 53.5 (35.3; 81.1) | 46.9 (42.3; 52) | 26.1 (25; 27.2) | 17.5 (16.5; 18.5) | 9 (8.3; 9.8) | 46.3 (43.5; 49.2) |
| Cardiovascular |  | 60.3 (48.3; 75.4) | 32.3 (29.9; 34.9) | 14.1 (13.3; 14.9) | 8.9 (8.4; 9.5) | 5 (4.4; 5.6) | 26.9 (24.7; 29.3) |
| Diabetes |  | 38.8 (33.4; 45.1) | 10.7 (9.9; 11.6) | 6.2 (5.8; 6.6) | 5.3 (4.9; 5.7) | 3.5 (3.2; 3.9) | 13.3 (12.2; 14.4) |
| Endocrine disorders |  | 6.9 (4.9; 9.7) | 3.9 (3.4; 4.4) | 3.4 (3.2; 3.6) | 3.7 (3.5; 3.9) | 3.1 (2.8; 3.4) | 7.8 (7.3; 8.3) |
| Blood disorders |  | 6.5 (3.1; 13.3) | 3.7 (2.4; 5.9) | 5.3 (4.3; 6.7) | 5.4 (4.3; 6.7) | 4.8 (3.6; 6.5) | 5.7 (4.5; 7.1) |
| Immunocompromised |  | 5.5 (4.2; 7.3) | 4.9 (4.3; 5.5) | 5.7 (5.3; 6.2) | 5.2 (4.8; 5.6) | 3.3 (3; 3.7) | 11.1 (10.3; 11.9) |
| Kidney disorders |  | 33.8 (20.5; 55.8) | 15.3 (12.9; 18.1) | 8.4 (7.6; 9.3) | 5.8 (5.4; 6.3) | 3.5 (3.1; 4) | 19.5 (17.6; 21.7) |
| Liver disorders | | 12 (9.5; 15.2) | 12 (6.8; 21.3) | 7.5 (6.3; 9) | 5.8 (5.4; 6.3) | 5 (4.6; 5.3) | 3.5 (2.9; 4.2) |
| Neurological disorders |  | 25.8 (14.1; 46.9) | 6.9 (5.2; 9.3) | 5 (4.4; 5.7) | 5.9 (5.4; 6.5) | 3.3 (2.8; 3.8) | 19.2 (16.9; 21.8) |
| Total population |  | 2.4 (2.1; 2.8) | 2.2 (2; 2.4) | 2.5 (2.3; 2.7) | 2.9 (2.6; 3.2) | 2.5 (2.1; 2.9) | 3.6 (3.1; 4) |
